# The Influence of Different Hand Paddle Size on 100-m Front Crawl Kinematics

**DOI:** 10.2478/v10078-012-0070-0

**Published:** 2012-10-23

**Authors:** Daniel López-Plaza, Fernando Alacid, Pedro A. López-Miñarro, José M. Muyor

**Affiliations:** 1 Department of Sport, Coaching and Exercise Science. University of Lincoln.; 2 Department of Physical Activity and Sports. University of Murcia.; 3 Department of Physical Education. University of Murcia.; 4 Department of Physical Education. University of Almería.

**Keywords:** Hand paddles, kinematic parameters, swimming performance

## Abstract

The purpose of this study was to determine the influence of different sizes of hand paddles on kinematic parameters during a 100 m freestyle swimming performance in elite swimmers. Nine elite swimmers (19.1 ± 1.9 years) completed three tests of 100 m without paddles, with small paddles (271.27 cm^2^) and with large paddles (332.67 cm^2^), respectively. One video camera was used to record the performance during the three trials. The mean swimming velocity, stroke rate and stroke length were measured in the central 10 meters of each 50 m length. The results showed that stroke length tended to increase significantly when wearing hand paddles (p < 0.05) during both the first and second 50 m sections whereas the increase in swimming velocity occurred only in the second 50 m (p < 0.05). Conversely, the stroke rate showed a slight decreasing trend with increasing paddle size. During the 100 m freestyle trial the stroke kinematics were changed significantly as a result of the increase in propelling surface size when hand paddles were worn.

## Introduction

During the past 30 years swimming performance has been studied from different perspectives. Some research has attempted to explain the influence of kinematic parameters on elite swimmers’ performance as well as their relationship over different competition distances (East, 1971; Craig et al., 1985; Keskinen et al., 1989; Arellano et al., 1994). Most of these studies have suggested that long stroke lengths are related to high average velocity of male elite swimmers. Nevertheless, the most significant variables influencing swimming velocity is stroke rate (Craig and Pendergast, 1979; Craig et al., 1985; Keskinen and Komi, 1993; Strzała et al., 2007).

In recent years coaches have used new training equipment in order to improve performance through the modification of stroke parameters. Several studies have evaluated the potential changes that auxiliary materials such as a swimming parachute or hand paddles can make to swimming performance (Llop et al., 2002; Gourgoulis et al., 2008a; 2009; Telles et al., 2011). Yet, the possibility of negatively affecting swimming technique through the use of this type of equipment is still a controversial issue among the swimming community, because this hypothesis has not been verified by experimental procedures (Maglischo, 2003). Hand paddles not only provide improvements in strength and aerobic performance, but have also been shown to have a diverse effect on swimming kinematics (Toussaint, 1990; Lauder and Newell, 2009). Because of the increase in propelling surface, swimming while wearing hand paddles tends to increase velocity and stroke length (Toussaint et al., 1991; Gourgoulis et al., 2006).

However, these investigations regarding hand paddles did not study the swimming performance in a realistic context. Rather they focused on analyzing the performance over non-competitive distances (25 m) and when wearing small buoys between the legs (Toussaint et al., 1991; Gourgoullis et al., 2008b; 2009). For this reason, the aim of this study was to analyze the influence of wearing different size hand paddles on kinematic parameters during a 100 m swimming performance. It was hypothesized that enlarging the propelling surface would substantially increase velocity and stroke length and decrease stroke rate during front crawl swimming.

## Methods

### Participants

Nine swimmers participated voluntarily in the study, 4 female (means ± standard deviations: age 17.9 ± 1.8 years, body height 1.69 ± 0.05 m, body mass 59.8 ± 3.1 kg) and 5 male (age 19.9 ± 2.3 years, body height 1.81 ± 0.06 m, body mass 80.6 ± 3.5 kg). They were elite national level swimmers (four Spanish national medallists and two international swimmers involved in the Spanish national swimming team) and had been training for at least 4 years (6–9 training sessions per week; 45 ± 15 km per week). The present study took place in July when national swimming competitions are usually scheduled, therefore, presumably swimmers reached an optimal fitness level. All participants were familiar with the use of different size hand paddles prior to the testing session. Each subject was required to abstain from hard training sessions for at least 24 hours before the test. The Institutional Ethical Committee of the University of Murcia approved the study and written informed consent form was obtained from all the swimmers before participation.

### Experimental design

In a random, counterbalanced order, participants completed three 100 m front crawl all out tests without hand paddles, with small and large hand paddles in a 50 m pool. A rest period of 30 minutes was given between trials. In the central 10 meters of both 50 m lengths (20–30 m and 70–80 m respectively) the kinematic parameters of each participant were measured. This avoided the influence of the start and the turn on kinematics. The swimmers covered the 100 m distance starting from the water and after a visual-acoustic signal.

For the determination of the central 10 meters, four external signals (buoys) were used as markers in the analysis of kinematics. The cross sectional areas of the two types of hand paddles (small paddles: 271.27 cm^2^; large paddles: 332.67 cm^2^) were analyzed by a computer program IMAGEJ (National Institutes of Health, USA). Every trial was recorded using a JVC Everio MG-155 video camera (Victor Company of Japan, Japan) at 30 frames/second. The images were analysed using Virtualdub software 1.8.8 by Avery Lee.

Kinematic data were obtained by one observer twice, with a two-month interval between observations. Internal consistency reliability for all the variables was determined using intraclass correlation coefficients (ICC 3.1), and the associated 95% confidence interval. The ICCs of the velocity and stroke rate were high, with a value of 0.99 in both cases.

### Data analysis

The stroke rate (SR) was calculated in Hz (cycles · s^−1^) by the number of frames taken to complete three stroke cycles within the central 10 meters of each 50 m length. The measurement started when the left hand of the swimmer first entered the water after the head had crossed the first reference line.

The average velocity (V) was obtained from the numbers of frames that each participant needed to cover the central 10 meters. The timing used the frames in which the head crossed reference lines at the start and end of the 10 meters. Finally, the stroke length (SL) was determined from the calculated stroke rate and average velocity: SL = V · SR^−1^ (m · cycle^−1^).

Descriptive statistics including means and standard deviations were calculated. The normality of the variables was analysed with the Shapiro - Wilk test. Since the distribution was not normal, comparisons between conditions were performed with Friedman repeated measures analysis of variance. If differences were detected, *post-hoc* comparisons were made using the Wilcoxon signed-rank paired *t*-test to determine where the differences had occurred. The data were analyzed using SPSS 15.0. The level of significance was set at *p* ≤ 0.05.

## Results

Table 1 presents mean velocity, stroke rate and stroke length for each type of paddle condition in the central 10 meters of each 50 m length. The ANOVA analysis revealed significant differences (*p* < 0.05) in the main effect of kinematic variables among the paddle conditions.

An increasing trend in the average velocity during the first 50 m was found, but no significant differences were observed between paddle conditions. Conversely, mean velocities in the second 50 m wearing both small and large paddles were significantly greater than without paddles (*p* < 0.01).

As for the stroke rate, no significant differences were detected either in the first 50 m or in the second 50 m. However, a decreasing trend was found as the size of the paddles was enlarged.

Stroke length wearing small paddles was significantly greater than without paddles in the first 50 m (*p* < 0.05). Stroke length wearing large paddles was significantly greater than without paddles during both the first 50 m (*p* < 0.01) and the second 50 m (*p* < 0.05).

**Figure 1:**
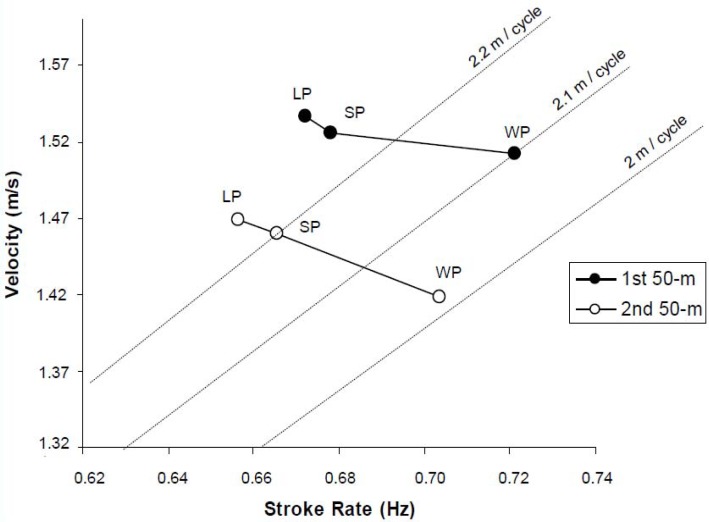
Velocity (m/s), stroke rate (Hz) and stroke length (m/cycle) relationship in each 50 m length during three different conditions: without paddles (WP), with small paddles (SP) and with large paddles (LP). Adapted from Craig et al. (1985).

Figure 1 shows the relationship between the three kinematic variables for each partial simultaneously. As the propelling surface was increased the tendency followed in the first 50 m moved diagonally towards the left part of the graph. The second 50 m followed the same tendency, but the trajectory depicted a more linear shape. In both 50 m lengths it meant an increase in velocity (up direction), a reduction in the stroke rate values (left direction) and an increase in stroke length (up and left diagonal direction).

## Discussion

The main purpose of the current study was to determine whether paddle size in a 100 m freestyle swimming performance influences kinematic parameters. The hypothesis that the increase in propelling surface would change significantly the kinematic parameters during front crawl swimming was supported. While comparing both 50 m lengths the kinematic parameters followed the same tendency: an increase in velocity and stroke length and a decrease in stroke rate. However, all values slightly decreased during the second 50 m length, perhaps due to fatigue. Moreover, the kinematic changes between the small and large paddle conditions were insignificant, probably, as a result of the minor differences in surface size (small paddles: 271.27 cm^2^; large paddles: 332.67 cm^2^). The most marked changes from the no-paddle condition were an increase in stroke length during both 50 m lengths when using large paddles, and an increase in velocity with increased size of the paddles in the second 50 m. By contrast, there were no significant changes in stroke rate, despite the fact that this parameter showed a slightly decreasing tendency, when propelling surface was enlarged. This perhaps meant that the temporal characteristics of the underwater stroke and the displacement of the hand were not significantly altered (Gourgoullis et al., 2008b) and the velocity increase was due to greater stroke length. However, this assumption questions the findings of other studies in stroke phase durations with hand paddles (Payton and Lauder, 1995; Lauder and Newell, 2009). They reported significantly longer pull time, especially in the upsweep phase, and less hand displacement when the propelling surface was increased. In an attempt to analyse phase durations (i.e. entry and catch, pull, push and recovery phases) and the pattern of arm coordination in front crawl swimming, Chollet et al. (2000) developed an index of coordination. Some studies (Gourgoulis et al., 2009; Telles et al., 2011) have focused particularly on the influence of swimming with hand paddles on this index. Although the duration of each phase is altered when the propelling surface is increased (Gourgoulis et al., 2009; Lauder and Newell, 2009) no significant differences in the pattern of arm coordination were found between both free swimming and hand paddle conditions (Gourgoulis et al., 2009; Telles et al., 2011).

The results of the comparison between free swimming and hand paddle swimming support the suggestion that hand paddles push backward greater masses of water because of the increase in propelling surface size in comparison with free swimming (Toussaint et al., 1991; Gourgoulis et al., 2009). This phenomenon would explain the greater stroke length and the higher mean swimming velocity observed in the present study. The propelling surface size is directly related to the propelling efficiency (Toussaint et al., 1991; Toussaint and Beek, 1992). At a given velocity, the energy expenditure decreases because of the increase in propelling surface. Therefore, swimming with hand paddles allows the maintenance of higher velocities than free swimming due to larger strokes per cycle without increasing the energy expenditure. This can be noticed in Figure 1, adapted from Craig et al. (1985), which simultaneously relates velocity to stroke rate and to stroke length. The larger the propelling surface, the higher the velocity in both 50 m segments as a result of a considerable increase in stroke length and an insignificant decrease in stroke rate.

Previous research (Toussaint et al., 1991; Gourgoulis et al., 2008a; 2009) analysed elite swimmers who were wearing different sized hand paddles over 25 m trials and similar results were obtained. The stroke rate decreased whereas stroke length values and velocity grew considerably when large paddles were worn. Gourgoulis et al. (2008b) reported decreases in stroke rate from 0.79 to 0.72 cycles per second and stroke lengths increases ranging from 1.72 to 1.94 meters per cycle when the propelling surface was enlarged. As for the velocity, Gourgoulis et al. (2009) found increases around 0.4 m/s in female swimmers. However, no real conditions were tested because trials were not conducted over a competitive distance and small buoys between the legs of the swimmers were used. Thus, the results from these studies must be treated with caution when comparing with the values from this research.

Studies which examined the effects of resisted swimming with parachutes also reported changes in kinematics (Llop et al., 2002; Gourgoulis et al., 2010). In these cases the velocity was reduced as a result of a decrease in both distance per cycle and cycle frequency. In contrast to swimming hand paddles, this kind of resisted swimming seems to have no significant effect on technique in comparison with free swimming (Gourgoulis et al., 2010). Lauder and Newell (2009) studied the changes in technique associated with the use of hand paddles by the analysis of the asymmetries in hand displacement. A tendency for more linear and shorter hand trajectories was found as the propelling surface was increased.

One of the limitations of this research was the fact that every swimmer was wearing the same paddles. It would have been more appropriate to use personal sized paddles depending on each swimmer’s hand measures, therefore, the paddles could have been made taking into consideration the same percentage increase in surface area for each participant. Another limitation of this study was that the number of participants, which was relatively small. Thus, it is suggested that the results from this investigation are treated with some caution, especially if comparing with others. Additionally, it would be interesting to compare our results with a larger sample in order to increase the power of statistical analysis.

## Conclusion

The stroke length was the kinematic parameter affected most strongly by the use of hand paddles. The velocity in the second 50 m wearing hand paddles was significantly higher than in the no paddle condition because of an increase in stroke length, whereas the stroke rate showed a slight negative trend with increasing paddle size. The present research has been the first to examine the use of hand paddles during race conditions on stroke kinematics. The influence of the start and turn was smaller than in previous studies because the tests were conducted over a 100 m distance in a 50 m swimming pool.

## Figures and Tables

**Table 1:** Mean ± standard deviation of velocity, stroke rate and stroke length during freestyle swimming without hand paddles, with small and large hand paddles

**Variables**	**First 50 m**			**Second 50 m**		

	Without paddles	Small paddles	Large padddles	Without paddles	Small paddles	Large padddles
	
Velocity (m/s)	1.52 ± 0.10	1.53 ± 0.11	1.54 ± 0.11	1.42 ± 0.12	1.46 ± 0.09**	1.47 ± 0.12**
Stroke rate (Hz)	0.73 ± 0.07	0.68 ± 0.05	0.67 ± 0.05	0.70 ± 0.07	0.67 ± 0.05	0.66 ± 0.05
Stroke length (m)	2.10 ± 0.20	2.25 ± 0.26*	2.29 ± 0.14**	2.03 ± 0.19	2.20 ± 0.17	2.25 ± 0.23*

* *p* < 0.05;

** *p* < 0.01 with respect to without paddles condition
